# Post-Transcriptional Regulation of Toll-Interacting Protein in the Intestinal Epithelium

**DOI:** 10.1371/journal.pone.0164858

**Published:** 2016-10-14

**Authors:** Yutaka Sugi, Kyoko Takahashi, Kenta Kurihara, Kazuaki Nakata, Hikari Narabayashi, Yuji Hamamoto, Makoto Suzuki, Masato Tsuda, Shigemasa Hanazawa, Akira Hosono, Shuichi Kaminogawa

**Affiliations:** 1 Department of Food Biochemistry and Biotechnology, College of Bioresource Sciences, Nihon University, Kanagawa, Japan; 2 Department of Applied Biological Science, College of Bioresource Sciences, Nihon University, Kanagawa, Japan; The University of Tokyo, JAPAN

## Abstract

Immune responses against gut microbiota should be minimized to avoid unnecessary inflammation at mucosal surface. In this study, we analyzed the expression patterns of Toll-interacting protein (Tollip), an inhibitor of TLRs and IL-1 family cytokine-related intracellular signaling, in intestinal epithelial cells (IECs). Comparable mRNA expression was observed in murine small and large IECs (S-IECs and L-IECs). However, Tollip protein was only detected in L-IECs, but not in S-IECs. Similar results were obtained in germ-free mice, indicating that L-IEC-specific TOLLIP expression does not depend on bacterial colonization. Next, to understand the mechanisms underlying the post-transcriptional repression of Tollip, 3´-UTR-mediated translational regulation was evaluated. The region +1876/+2398 was responsible for the repression of Tollip expression. This region included the target sequence of miR-31. The inhibition of miR-31 restored the 3´-UTR-meditaed translational repression. In addition, miR-31 expression was significantly higher in S-IECs than in L-IECs, suggesting that miR-31 represses the translation of Tollip mRNA in S-IECs. Collectively, we conclude that the translation of Tollip is inhibited in S-IECs, at least in part, by miR-31 to yield L-IEC-specific high-level expression of the Tollip protein, which may contribute to the maintenance of intestinal homeostasis.

## Introduction

Although gut microbiota is immunologically “non-self” to the host, the host immune system does not exclude them completely and resulting in a symbiotic relationship. The gut microbiota is a complex of over 1000 species and ~100 trillion bacterial communities. The mammalian intestine serves as a convenient habitat because of its temperature, anaerobic conditions, and abundance in nutrients. There is an increasing amount of evidence on the benefits of the symbiosis that the host enjoys. Actually, the significance of gut microbiota for the host’s health has attracted considerable attention. For example, the importance of gut microbiota, especially for its essential contributions to the host’s metabolism, has been reported[1, 2]. In addition, gut microbiota is also known as one of the key modulators of both the host’s local and systemic immune responses[3, 4].

A single layer of intestinal epithelial cells (IECs) forms a physical barrier by separating the luminal contents, including gut microbiota, from the inside of the body. IECs are continuously exposed to a variety of pathogen-associated molecular patterns (PAMPs) derived from gut microbiota; however, homeostasis is usually maintained by regulating immune responses through pattern recognition receptors (PRRs) such as Toll-like receptors (TLRs)[5–7]. On the other hand, excessive IEC responses to gut microbiota could cause inflammatory or autoimmune diseases[8, 9]. Hence, the balance between the pro-inflammatory and the tolerogenic immune responses to gut microbiota has been understood to support intestinal homeostasis.

Some anti-inflammatory molecules produced by IECs that maintain homeostatic conditions, e.g. preventing negative consequences such as chronic immune activation and inflammation, have been reported. For instance, mucins and anti-microbial peptides are secreted by IECs to prevent adhesion of commensal bacteria to the epithelial surface[10, 11]. Intestinal alkaline phosphatase (iAP) is also secreted by IECs to detoxify harmful bacterial LPS[12, 13]. In addition to such secretory molecules, the regulation of TLR and TLR-related signaling molecule expression is reported as another mechanism by which IECs prevent excessive inflammation[14–17]. Notably, the regulation is bidirectional, and expression of these molecules is in turn partially regulated by gut microbiota; for example, TLR-MyD88-dependent expression of specific TLRs is differently affected by gut microbiota[18, 19]. In this study, we focused on Toll-interacting protein (Tollip)[20], an inhibitor of TLRs and IL-1 family cytokine-related intracellular signaling, in IECs. Tollip is expressed in IECs at relatively higher levels than in monocytes[21]. Tollip knockout mice do not develop spontaneous colitis, but are susceptible to dextran sodium sulfate (DSS)-induced colitis[22]. In addition, the severity of spontaneous colitis in IL-10 knockout mice is increased by Tollip deficiency. Therefore, Tollip is thought to play an important role in preventing harmful inflammatory responses. However, the underlying gene expression mechanisms are not well understood. In this study, we have shown that besides being regulated at the transcriptional level[23], the expression of Tollip is strongly regulated at the post-transcriptional level, at least in part, via miR-31. Micro RNAs (miRNAs) are small noncoding RNAs that are generally known to suppress the expression of specific mRNAs by binding to their 3´ untranslated region (UTR). Of the thousands of miRNAs identified so far, miR-31 is an evolutionarily highly conserved miRNA, regulating the expression of various genes involved in the proliferation, differentiation, and motility of cells[24–26]. miR-31 is also a biomarker of autoimmune diseases and cancer[27–31]. Here we report the involvement of this miRNA in the post-transcriptional regulation of the Tollip gene.

## Materials and Methods

### Mice

BALB/c mice were purchased from CLEA Japan (Tokyo, Japan) and bred under conventional (CV) conditions. Germ-free (GF) BALB/c mice were bred and kept in our GF facility at College of Bioresource Sciences, Nihon University. Mice were maintained in a temperature-controlled room with a 12 h light/dark cycle with free access to food and water. Female mice were studied at 10–12 weeks of age. All experiments were approved by the Nihon University Animal Care and Use Committee (Permit Number: AP12P028) and conducted in accordance with the guidelines.

### Preparation of IECs

Small and large intestines (the colon and rectum, but not the cecum) were surgically excised from mice. The small intestines were divided into five pieces of equal length, and the first (proximal), the third (medial), and the fifth (distal) parts were taken. The intestines were washed with Ca^2+^- and Mg^2+^-free Hanks’ balanced salt solution (HBSS) (Sigma, St. Louis, MO) containing 5% fetal calf serum (FCS) (Biowest, Nuaillé, France) to remove the luminal content. Single cell suspensions of IECs were prepared as described previously[23], with a few modifications. After treatment with dispase (BD Biosciences, Franklin Lakes, NJ), lymphocytes were depleted from the IEC fraction using Dynabeads M-280 or M-450 Streptavidin (Invitrogen, Carlsbad, CA) and biotin-conjugated anti-CD45 antibody (eBioscience, San Diego, CA). IEC purity was assessed by flow cytometry and staining with phosphatidylethanolamine-Cy-7-labeled anti-CD45 antibody (eBioscience) and propidium iodide (PI) (Sigma); CD45 cells were less than 3% of the total number of PI negative live cells. IECs pooled from 4–7 individuals of mice were used for each experiment.

### Cell culture

The murine rectal carcinoma line CMT-93 was purchased from DS Pharma Biomedical (Osaka, Japan). The cells were cultured in Dulbecco’s Modified Eagle’s Medium (Nissui Pharmaceutical, Tokyo, Japan) supplemented with 10% (v/v) FCS, 100 U/mL of penicillin (Meiji Seika Pharma, Tokyo, Japan), 100 μg/mL streptomycin (Meiji), and 5 × 10^−5^ M 2-mercaptoethanol at 37°C in a humidified incubator with 5% CO_2_.

### qPCR

For the quantitative analysis of mRNA and miRNA expression, total RNA was prepared from IECs using High Pure RNA Isolation Kit and High Pure miRNA Isolation Kit, respectively (Roche Diagnostics GmbH). Total RNA was then reverse-transcribed using Super-Script II Reverse Transcriptase (Invitrogen) and oligo(dT)_12-18_ primers (Invitrogen) for mRNA quantification or Universal cDNA Synthesis Kit (Exiqon, Vedbaek, Denmark) for miRNA quantification.

The resulting cDNA was quantified by real-time PCR with a LightCycler 480 (Roche Diagnostics GmbH) using the SYBR Green I master reagent (Roche Diagnostics GmbH). mRNA expression was normalized to the expression of Gapdh. For miRNA expression, values obtained using equal amounts of RNA were used without normalization. Normalization to U6 or RNU1A1, both of which are usually used as references in such experiments, was not suitable here, as the expression of these miRNAs changed remarkably depending on the differentiation status of IECs. Information on primers is listed in S1 Table. For the quantification of miR-31, mmu-miR-31-5p LNA™ PCR primer set, UniRT (Exiqon, Product No. #205159) was used.

### Western blotting

IECs were homogenized in cell-lysis buffer with a 21-gauge needle and incubated on ice for 30 min[23]. After centrifugation at 4°C, 20,000 × g, for 10 min, the supernatants were collected. The protein content of the lysates was measured by the Pierce™ protein assay kit (Thermo Scientific, Waltham, MA), and equal amounts of protein were analyzed by immunoblotting using anti-TOLLIP (ab62578, Abcam, Cambridge, UK) and anti-β-ACTIN (Abcam) antibodies.

### Plasmid construction

The full length 3´ UTR of mouse Tollip mRNA (RefSeq ID: NM_023764.3) was obtained by PCR using reverse transcripts of large IECs as a template. The amplified product was inserted into the pGL3-Control Vector (Promega, Madison, WI) between BamHI and blunted XbaI (Takara Bio, Shiga, Japan) sites downstream of the luciferase gene and verified for the sequence. The resulting plasmid was named Luc-mTollip_3´-UTR1-2782. Nucleotide numbers 1 and 2782 represent the first nucleotide just behind the stop codon and the last nucleotide just upstream the poly(A)-tail, respectively.

Luc-mTollip_3´-UTR Δ1–1604, Δ160–2398, and Δ901–1722 were constructed by self-ligation after the digestion of Luc-mTollip_3´-UTR 1–2782 with restriction enzymes XbaI, EcoT22I, and PstI (Takara), separately. Luc-mTollip_3´-UTR Δ550–1876 was constructed by self-ligation of the blunt-ended product treated with Bpu10I (New England Biolabs, Ipswich, MA). For the construction of Luc-mTollip_3´-UTR Δ1–2572 and Δ1–2646, each deletion fragment of the 3´-UTR of the mouse Tollip gene was obtained by PCR and inserted into the BamHI and XbaI sites. To construct Luc-mTollip_3´-UTR Δ1–1172, a PCR fragment corresponding to the 3´-UTR 1173–1604 was inserted into Luc-mTollip_3´-UTR Δ1–1604 after XbaI digestion.

### Transient gene expression and reporter gene assays

CMT-93 cells were transfected with 0.7 μg of each constructed plasmid, 5 ng of phRL-CMV (Promega), and 2.1 μl of X-tremeGENE HP DNA Transfection Reagent (Roche Diagnostics GmbH), following the manufacturer’s instructions. Twenty-four hours after transfection, luciferase activity was measured using the dual-luciferase assay kit (Promega), according to the manufacturer’s instructions. Data shown are mean ± SD values of three independent experiments, and are reported as fold-induction to cells transfected with Luc-mTollip_3´-UTR1-2782.

For the miRNA inhibition assay, each miRNA-specific inhibitor or control inhibitor was transduced into CMT-93 cells at the concentration of 5 nM with the reporter plasmid Luc-mTollip_3´-UTR1-2782, using X-tremeGENE siRNA Transfection Reagent (Roche Diagnostics GmbH). After culturing for 24 h, the cells were lysed to measure luciferase activity, as described earlier. Data shown are mean ± SD values of two or three independent experiments and expressed as values relative to the control without inhibitors.

### Northern blotting and RNase H assay

RNAs (1.5 μg) were separated by electrophoresis on a formaldehyde / agarose gel in 1 × MOPS buffer (Nacalai Tesque, Kyoto, Japan) and blotted onto a positively charged nylon membrane (GE Healthcare UK Ltd., Buckinghamshire, England). Hybridization was performed using DIG Northern Starter Kit (Roche Diagnostics GmbH), according to the manufacturer’s instructions. DIG-labeled RNA probes targeting the coding region and the 3´-UTR of the mouse Tollip gene were constructed by T7 RNA polymerase reaction.

RNase H assay was performed as described by Wakiyama et al.[32] with a few modifications. Two micrograms of total RNA was mixed with 100 pmol of oligonucleotides targeting the region close to boundary between the CDS and the 3´-UTR of Tollip, in combination with 500 ng of oligo(dT)_12-18_ (Invitrogen), or 100 pmol of oligonucleotides targeting the region close to boundary between the 5´-UTR and CDS. Each oligo DNA and RNA was mixed and denatured for 5 min at 70°C and annealed for 10 min at 37°C. They were then incubated with 5 U of RNase H (New England Biolabs) in 1 × RNase H buffer for 60 min at 37°C.

## Results

**Fig 1:**
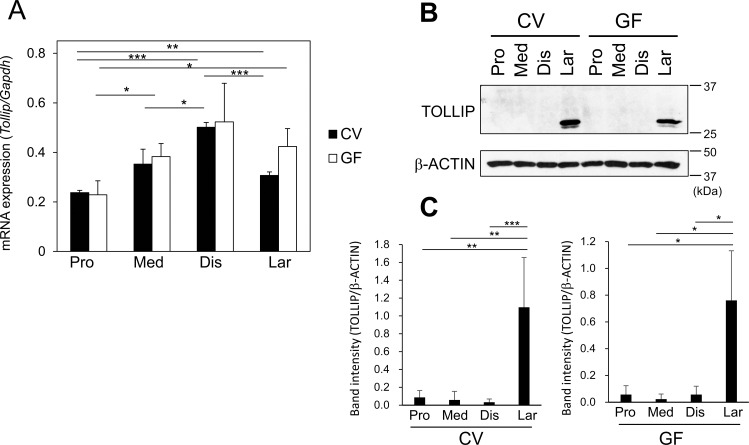
Tollip gene expression in IECs is strongly regulated at the post-transcriptional level. (A) RNA samples from proximal (Pro), medial (Med), and distal (Dis) S-IECs and L-IECs (Lar) of CV and of GF mice were analyzed by quantitative RT-PCR. The values were normalized to that of Gapdh. Data are presented as mean ± SD values of three independent experiments. Differences were analyzed using Student’s t-test. * *P* < 0.05, ** *P* < 0.005, *** *P* < 0.0005. (B, C) IECs were isolated from the indicated regions of the intestine. The total cell lysate was analyzed by immunoblotting with anti-TOLLIP and anti-β-ACTIN antibodies. Representative blots of six (CV) or four (GF) independent experiments and mean ± SD of the relative band intensities normalized to those of β-ACTIN are shown in (B) and (C), respectively. Differences were analyzed using Student’s t-test. * *P* < 0.05, ** *P* < 0.01, *** *P* < 0.005.

First, we analyzed the expression of the Tollip mRNA in IECs. For the preparation of IEC samples, we divided the small intestine into five parts of equal length and then used the proximal, medial, and distal parts for the analysis because of the difference in their bacterial density[33, 34]. Tollip mRNA expression gradually increased from the proximal to the distal part of the intestine in small IECs (S-IECs). In large IECs (L-IECs), its expression level was comparable with that in medial S-IECs. Furthermore, such an expression pattern in CV mice was also observed in GF mice (Fig 1A). These data suggest that Tollip mRNA expression is not affected by bacterial colonization. Next, we checked Tollip expression at the protein level. Immunoblotting analysis showed that TOLLIP was abundantly expressed in L-IECs but barely detected in proximal, medial, and distal S-IECs in both CV and GF mice (Fig 1B). The protein expression profile did not correlate with the mRNA expression patterns shown in Fig 1A. These results indicate that Tollip expression is strongly regulated at the post-transcriptional level in a manner independent of gut microbiota.

The UTR of mRNA is known to influence its translational efficiency[35–37]. The stem-loop-like structure on both the 5´ and the 3´ region is recognized by a specific RNA-binding protein, depending on their sequence or structure, thereby controlling its expression through subcellular localization. The other mechanism of UTR-mediated translational control is mediated by miRNA. miRNA binds to the specific RNA through the sequences in both the 5´-[38] and the 3´-UTR[39] and controls the translation of the targeted RNA. Tollip mRNA has relatively a longer 3´-UTR; it is approximately 3 times longer than the protein coding region (823 nt CDS vs. 2716 nt 3´-UTR). Therefore, we examined whether the translation of Tollip mRNA is regulated through its 3´-UTR.

**Fig 2:**
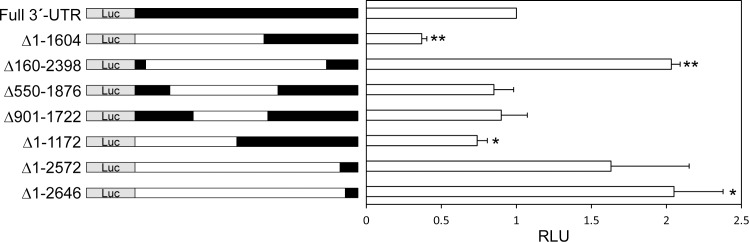
The region +1876/+2398 in the 3´-UTR is responsible for the translational repression of the Tollip gene. The *cis*-acting region in 3´-UTR of the Tollip gene is determined by a reporter gene assay using a series of deletion constructs in CMT-93 cells. Luciferase activity relative to that of WT (full-length of 3´-UTR) is shown. The results are expressed as mean ± SD values of three independent experiments. Nucleotide numbers are counted from the first nucleotide of the 3´-UTR by +1. Differences were analyzed using Student’s t-test. * *P* < 0.05, ** *P* < 0.0005. RLU, relative light units.

**Fig 3:**
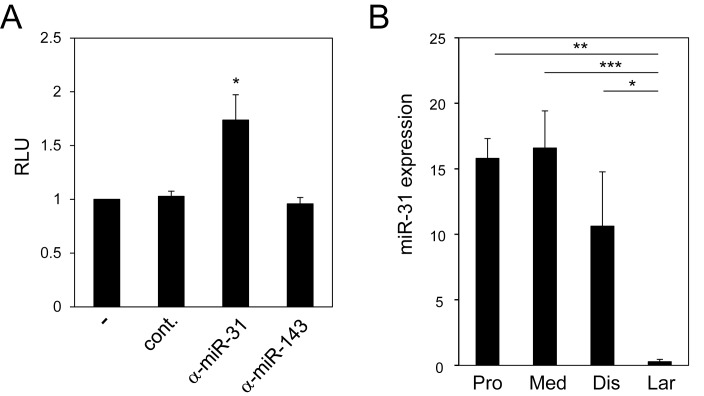
miR-31 suppresses the translation of the Tollip gene in S-IECs. (A) CMT-93 cells were co-transfected with a reporter plasmid carrying the full-length 3´-UTR of the Tollip gene and miR-31 inhibitor (α-miR-31), miR-143 inhibitor (α-miR-143) or negative control (cont.). The results are expressed as mean ± SD values of three independent experiments. Differences were analyzed using Student’s t-test. * *P* < 0.05. (B) Quantitative analysis of miR-31 expression in S-IECs (Pro, Med, Dis) and L-IECs (Lar) of mice. The results are expressed as mean ± SD values of three independent experiments. Differences were analyzed using Student’s t-test. * *P* < 0.05, ** *P* < 0.01, *** *P* < 0.005.

To address this, we performed the luciferase assay using the murine epithelial cell line CMT-93 and assessed the effect of the 3´-UTR of the Tollip gene on its translation. Deletion of nt 160–2398, but not of nt 550–1876, 901–1722, 1–1604, or 1–1172, significantly increased the luciferase activity, indicating that the region nt 1876–2398 suppresses the Tollip gene expression (Fig 2). Consistently, deletion of nt 1–2572 or 1–2646 also increased the luciferase activity. McKenna et al. analyzed miRNA expression in murine IECs and found that expression levels of some miRNAs were significantly different between S-IECs and L-IECs[40]. Furthermore, based on miRBase.org[41] and microRNA.org[42], there were a couple of candidate miRNAs that bind to the nt 1876–2398 region of the Tollip gene. Among the candidates, two miRNAs, miR-31-5p (miR-31) and miR-143-3p (miR-143), were expressed at much higher levels in S-IECs than in L-IECs[40]. Inhibitors of these miRNAs were introduced into CMT-93 cells to analyze the effect of these miRNAs on the Tollip gene expression. The inhibition of miR-31 upregulated luciferase activity, while miR-143 had no effect (Fig 3A). Next, miR-31 expression in S-IECs and L-IECs was measured by RT-qPCR (Fig 3B). miR-31 expression was approximately 50-80-fold higher in proximal and medial S-IECs than in L-IECs. miR-31 expression in distal S-IECs was also significantly higher than in L-IECs. The difference among the IECs from the proximal, medial, and distal regions of the small intestine was not statistically significant. These results show that miR-31 is expressed at high levels and inhibits the translation of Tollip mRNA through binding to nt 1876–2398 of the 3´-UTR in S-IECs, but not in L-IECs.

**Fig 4:**
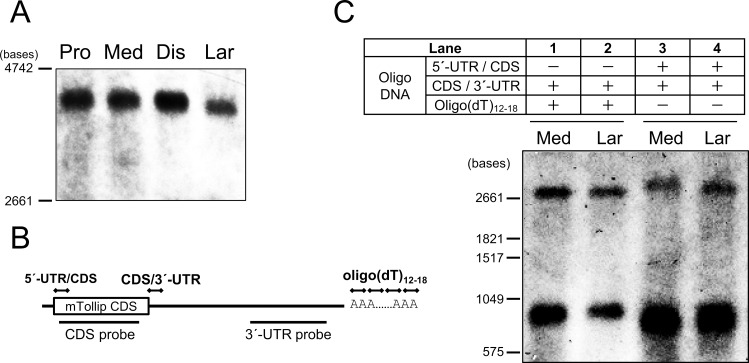
Tollip mRNA in L-IECs has a shorter poly(A)-tail. (A) Tollip mRNA in proximal (Pro), medial (Med), and distal (Dis) S-IECs and L-IECs (Lar) of CV mice was analyzed by northern blotting. The blots are representative of three independent experiments. (B) Schematic drawing of oligo DNAs and detection probes used in the assay. (C) The total RNA from medial S-IECs (Med) or L-IECs (Lar) was incubated with oligo DNA, designed as indicated in (B), and RNase H. After 1 h of incubation, the samples were analyzed by northern blotting to detect Tollip mRNA fragments using the illustrated probe. The blots are representative of two independent experiments.

Since the recovery of luciferase activity by miR-31 inhibition was relatively modest, another mechanism is possibly involved in the UTR-mediated regulation of Tollip mRNA translation. Furthermore, the molecular mass of Tollip mRNA in L-IECs seemed to be smaller than that in S-IECs according to northern blotting (Fig 4A), suggesting the involvement of different UTR structures in translational efficiency. To explain why Tollip mRNA in L-IECs is smaller, RNase H assays were conducted with oligo DNAs designed to recognize the boundary region between the 5´-UTR and the CDS or poly(A)-tail, each of which was combined with the oligo DNA recognizing the boundary between 3´-UTR and CDS. The mixture of two detection probes shown in Fig 4B was used for the assay. The bands represent CDS with 5´-UTR (lower band in lanes 1 and 2), CDS alone (lower band in lanes 3 and 4), 3´-UTR alone (upper band in lanes 1 and 2), and 3´-UTR with poly(A)-tail (upper band in lanes 3 and 4), respectively. The upper band in lane 3 (medial S-IECs) had a higher molecular mass than that in lane 4 (L-IEC), while there was no difference between S-IECs and L-IECs regarding other bands. The results indicate that the poly(A)-tail of the Tollip mRNA was shorter in L-IECs than in S-IECs.

## Discussion

In this study, we have shown that Tollip expression is strongly regulated at the post-transcriptional level and, thus, TOLLIP protein is expressed at much higher levels in L-IECs than in S-IECs. Enhanced expression of Tollip in IECs has been reported previously[43]. It has been reported that mRNA expression in human biopsy samples is enhanced from the proximal to the distal part of the intestine and that this reflects bacterial density, but is not determined at the protein level. In addition, it has also been reported in various human IEC lines that the expression of Tollip is upregulated by stimulation of the TLR ligand. However, Tollip expression in normal IECs in vivo has not been reported. We showed that Tollip mRNA expression in L-IECs was comparable to the medial S-IECs and that TOLLIP protein is abundantly expressed in L-IECs but barely detected in S-IECs, and that it is not dependent on bacterial colonization, i.e. TLR-mediated stimuli and bacterial metabolites, because similar L-IEC-specific TOLLIP expression has been observed in GF mice. However, the upregulation of Tollip mRNA by gut microbiota was reported recently by Brandão et al. in mid small intestinal tissues from CV and GF mice[19]. The difference may be attributed to the different mouse strains used (they used C57BL/6 mice, while BALB/c mice were used in our study) and/or the samples used for the analysis (they used whole tissues, while we used purified IECs). As the interaction between gut microbiota and TLR-related molecules have been reported[17–19], the role of TOLLIP in the large intestine which is inhabited by a huge number of commensal bacteria remain to be elucidated.

Tollip regulates the severity of DSS-induced and spontaneous colitis in IL-10 KO mice[22]. Because more gut microbiota inhabit the large intestine than the small intestine, L-IEC-specific expression of TOLLIP may be a strategy of avoiding an excessive response to gut microbiota by suppressing TLR signaling. Interestingly, TOLLIP protein expression seems to be different depending on mouse strains (S1 Fig). The finding that TOLLIP is expressed at a lesser level in L-IECs of C57BL/6 mice than in those of BALB/c mice may reflect the observation that C57BL/6 mice are more sensitive to DSS-induced colitis than BALB/c mice.

In addition to inhibiting TLR signaling, several reports show that TOLLIP binds to ubiquitinated proteins, for instance, TGFβR1[44] and IL-1RI[45], to control inflammatory responses[46, 47]. Recently, Tollip has been identified as the human homologue of the yeast coupling of ubiquitin conjugation to endoplasmic reticulum (ER) degradation protein 5 (Cue5), which functions as a ubiquitin-Atg8 (yeast counterpart of human LC3) receptor and regulates selective autophagy[48]. Autophagy in IECs has a critical role in bacterial clearance[49–51] and regulates inflammatory bowel disease[52, 53]. These suggest that Tollip is also involved in autophagy and thereby suppressing excessive inflammation in L-IECs. Related to autophagy, an increasing body of evidence shows that ER stress-sensing molecules are also important in the regulation of colitis because the abnormalities of such molecules result in impaired IEC maturation[54–57]. Tollip may prevent such ER stress-related colitis through the removal of accumulated abnormal proteins by autophagy. Therefore, constitutive expression of TOLLIP in L-IECs may crucially regulate not only TLR-signaling but other cellular responses important for homeostasis as well.

In contrast to L-IECs, TOLLIP is hardly expressed in S-IECs; S-IECs may be more sensitive to TLR-mediated stimulation by gut microbiota. Anti-microbial peptides and immunoglobulin (Ig) A are produced predominantly in S-IECs. TLR signaling is required for anti-microbial peptide expression, which is responsible for spatial segregation in the small intestinal mucosa[11]. In addition, recognition of gut microbiota by IECs via TLRs also accounts for the production of B cell-activating factor (BAFF)[58] and a proliferation-inducing ligand (APRIL)[59] necessary to promote IgA-producing plasma cell differentiation in the lamina propria.

We found that miR-31 represses the translation of Tollip. miR-31 is predominantly expressed in S-IECs. Other groups also showed higher expression of miR-31 in S-IECs than in L-IECs[40, 60]. miRNAs in IECs play a role in cellular differentiation, e.g. M-cells[61] and goblet cells, helminth infection[62], flora composition[63], lipid metabolism, and inflammatory response[40]. miR-31 is coded on chromosome 4 near the region with the ifn α/β genes; however, ifn α/β genes are not transcribed constitutively in S-IECs or in L-IECs[64], suggesting a specific transcriptional activation of miR-31 expression in S-IECs. This regulation is not dependent on inflammation because miR-31 expression was not different in DSS-induced colitis[60]. McKenna et al. showed that A20, one of the target genes of miR-31, is up-regulated in dicer1-deficient mice[40]. Because A20 is also known to negatively regulate TLR signaling, it is thought that a higher expression of miR-31 leads to the reduced expression of these negative regulators, so as to increase the sensitivity to bacterial stimulation in S-IECs.

We also found a difference in Tollip mRNA poly(A)-tail length in S-IECs and L-IECs, which may contribute to the different translation efficiency between these cells. The poly(A)-tail of mRNAs plays an important role in translation initiation, and stability[65, 66]. Translational repression of target mRNA by miRNA is known to be usually mediated by deadenylation[32, 67]. In contrast, as shown in Fig 3B, despite the microRNA-mediated translational repression, Tollip mRNA in S-IECs has a longer poly(A)-tail than that in L-IECs. However, a recent study showed such a poly(A)-tail-mediated translational enhancement is preferentially observed in immature zygotic cells, but not in somatic cells[68]. Therefore, it is still unclear whether such translational control occurs in IECs. There is a possibility that the longer poly(A)-tail in S-IECs is linked to the lower translation of Tollip mRNA because of inefficient nuclear-cytoplasmic transport. Actually, poly(A)-tail could induce nuclear export of the mRNA but transcript carrying very long poly(A)-tail approximately over 250 nt failed in the transportation to the cytoplasm and retained in the nuclei in Xenopus oocytes [69]. In conclusion, our data suggest that Tollip expression is strongly regulated at the post-transcriptional level. L-IEC-specific Tollip expression may contribute to the homeostasis at the mucosal surface and maintain a symbiotic relationship between the host and gut microbiota.

## Supporting Information

S1 Fig Strong TOLLIP expression in L-IECs is detected in BALB/c mice but not in C57BL/6 mice. Lysates from each IECs of CV C57BL/6 and BALB/c mice were immunoblotted with antibodies against TOLLIP (top) and β-ACTIN (bottom). The representative blots of three independent experiments were shown.
(TIF)

S1 Table Primers used in this study. Nucleotide sequences of the primers are shown.
(XLSX)
