# Supplementary figures and images for: Post-Transcriptional Regulation of Toll-Interacting Protein in the Intestinal Epithelium
Source: PLoS One. 2016 Oct 14;11(10):e0164858. doi: 10.1371/journal.pone.0164858 (PMC5065231; doi:10.1371/journal.pone.0164858)

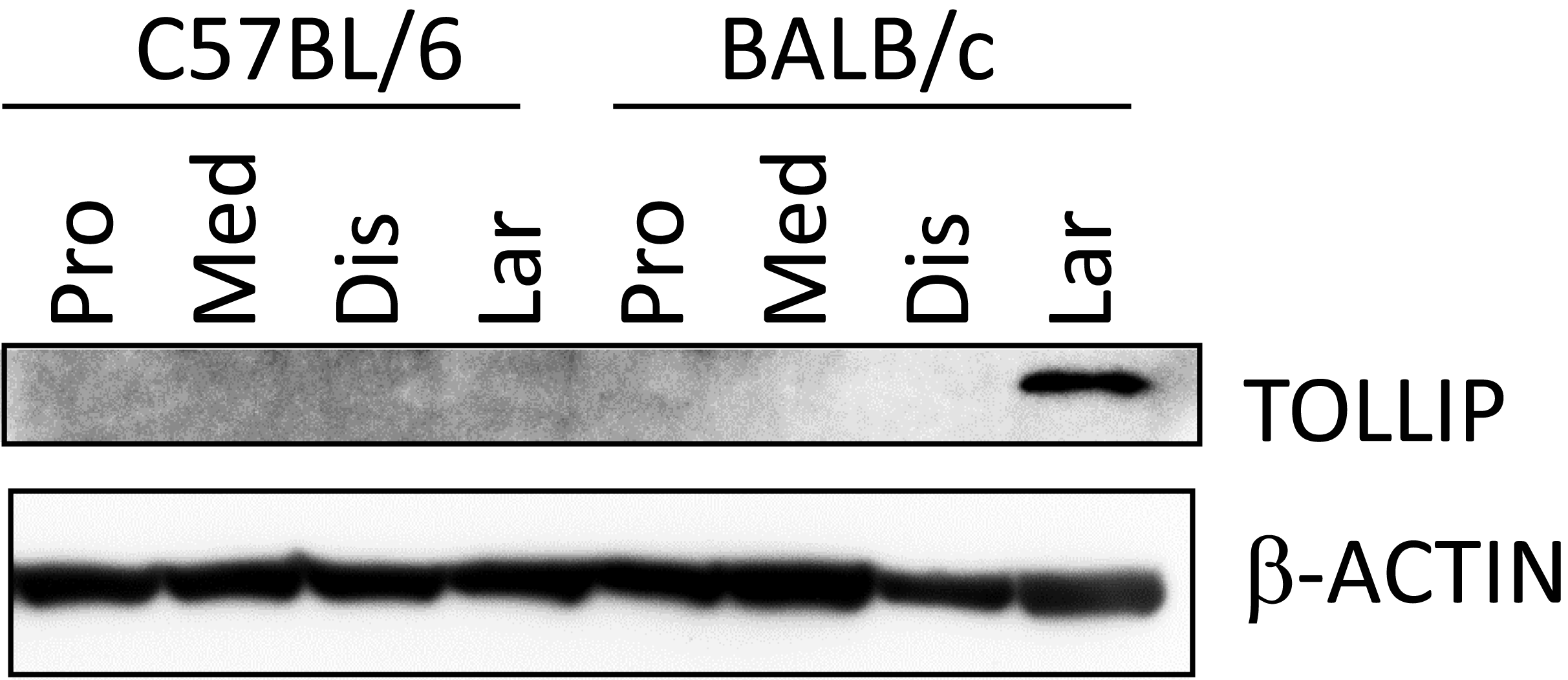

Supplement: S1 Fig — Lysates from each IECs of CV C57BL/6 and BALB/c mice were immunoblotted with antibodies against TOLLIP (top) and β-ACTIN (bottom). The representative blots of three independent experiments were shown. (TIF) [file pone.0164858.s001.tif]
